# Comparative study of Pd-, Au-, and Cu-based nanostructured electrodes synthesized in deep eutectic solvents: linking nucleation mechanisms to electrochemical DNA sensing performance

**DOI:** 10.1039/d6ra02774a

**Published:** 2026-07-02

**Authors:** Phuong Dinh Tam, Dao Vu Phuong Thao, Nhi Phuong Hien, Dinh Van Tuan, Nguyen Thi Nguyet

**Affiliations:** a Faculty of Materials Science and Engineering, Phenikaa School of Engineering, Phenikaa University Hanoi 10000 Vietnam tam.phuongdinh@phenikaa-uni.edu.vn phuongdinhtam@gmail.com; b Department of Chemistry, Hartwick College Oneonta NY 13820 USA; c Electric Power University Hanoi Vietnam; d Faculty of Chemical and Environment, Hung Yen University of Technology and Education Hung Yen Vietnam

## Abstract

This work presents a systematic comparison of the nucleation mechanisms of Pd, Au, and Cu in deep eutectic solvents and their direct effect on electrochemical DNA sensing performance. Chronoamperometric analysis revealed progressive nucleation for Cu, mixed nucleation for Au, and instantaneous nucleation for Pd. The distinct nucleation behaviors significantly influenced the surface morphology and electrochemical characteristics of the deposited electrodes. Accordingly, the sensitivities of Cu-, Au-, and Pd-based sensors were determined to be 80.48, 163.35, and 318.54 µA nM^−1^ cm^−2^, respectively, and the corresponding limits of detection (LOD) were 1.97 × 10^−12^ M, 1.17 × 10^−12^ M, and 0.73 × 10^−12^ M. All sensors exhibited a wide linear detection range from 1.0 × 10^−12^ to 3.0 × 10^−10^ M, excellent reproducibility (RSD < 3.2%), good stability, and high selectivity toward complementary DNA sequences. Practical applicability was validated through the detection of PCR-amplified DNA extracted from the sputum samples of patients with tuberculosis, showing satisfactory recovery and reliability in complex biological matrices.

## Abstract 2

This work presents a systematic comparison of the nucleation mechanisms of Pd, Au, and Cu in deep eutectic solvents and their direct effect on electrochemical DNA sensing performance.

## Graphical abstract

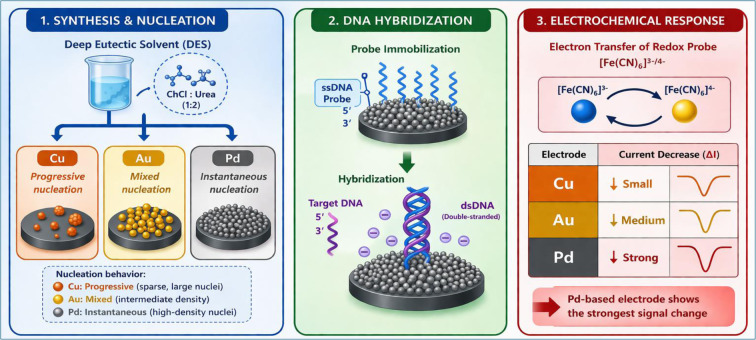

## 1 Introduction

The rapid and sensitive detection of specific DNA sequences plays a crucial role in many fields, including clinical diagnostics, environmental monitoring, food safety, and pathogen identification.^1^ Traditional molecular techniques such as polymerase chain reaction (PCR),^2^ fluorescence-based assays,^3^ and gel electrophoresis^4^ provide reliable results but often require sophisticated instrumentation, time-consuming procedures, and well-equipped laboratories.^5^ Electrochemical DNA biosensors^6,7^ have emerged as attractive alternatives because of their high sensitivity, rapid response, low cost, and potential for miniaturization and point-of-care analysis. Hybridization-driven biosensing strategies have also been widely explored in electrochemical biosensors and DNA-assisted immunosensors owing to their excellent specificity and signal amplification capability.^8^

Electrochemical DNA sensors generally operate through the immobilization of probe DNA on the electrode surface, followed by hybridization with complementary target DNA.^9^ This hybridization changes the interfacial electron-transfer behavior of redox probes, which can be monitored through electrochemical techniques including cyclic voltammetry,^6,10^ differential pulse voltammetry,^11,12^ squarewave voltammetry,^13,14^ and electrochemical impedance spectroscopy.^6,10,15^ The analytical performance of these sensors strongly depends on the physicochemical properties of the electrode surface, particularly the electroactive surface area, surface roughness, and electron-transfer kinetics,^16^ which play critical roles in determining the electrochemical response efficiency during DNA hybridization.

Metal nanostructures such as Au,^17,18^ Pd^19,20^ Cu,^21–23^ Ni^24^ have attracted considerable attention as electrode materials for biosensing fabrications to improve these properties. Gold has been extensively used in electrochemical biosensors because of its excellent conductivity, chemical stability, and strong affinity toward biomolecules.^18,24,25^ Palladium has also demonstrated remarkable high electroactive surface area, electrocatalytic activity, and favorable electron-transfer properties.^19,26^ Although less noble than Au and Pd, copper offers advantages in terms of cost and ease of electrodeposition^21^ and has been increasingly explored for electrochemical biosensor applications.

In parallel with material selection, the electrolyte and synthesis environment play equally critical roles in determining electrode morphology and DNA sensor performance.^27,28^ Deep eutectic solvents (DESs) have recently emerged as environmentally friendly and versatile alternatives to conventional aqueous or acidic electrolytes for metal electrodeposition.^29,30^ DESs typically possess wide electrochemical windows, high ionic conductivity, low volatility, and excellent metal salt solubility.^31,32^ Their relatively high viscosity and reduced ion diffusivity enable controlled nucleation and growth, allowing the formation of uniform, high-surface-area nanostructures.^29^ These characteristics make DESs particularly attractive for fabricating nanostructured sensing interfaces through green and sustainable routes. Compared with conventional acidic electrolytes, DESs exhibit negligible vapor pressure, lower toxicity, and reduced generation of hazardous waste, making them more environmentally benign media for electrodeposition. Although DES has been extensively investigated to nanomaterial synthesis for catalysis,^33^ water treatment,^34^ corrosion protection^30^ and electrochemical sensor,^35^ its applications in DNA sensor fabrication is still at an early stage.

Despite the growing interest in metal electrodeposition in DESs for DNA biosensor applications, most previous studies focused on the deposition behavior or electrochemical properties of individual metals. A systematic comparison of different metal electrodes synthesized under identical DES conditions and their influence on electrochemical DNA sensing performance is lacking. In particular, the relationship between nucleation mechanisms during electrodeposition and the analytical performance of DNA biosensors has not been clearly established.

In this work, we present a comparative study of nanostructured Cu, Au, and Pd materials synthesized by electrodeposition in a DES for electrochemical DNA sensing applications. The nucleation and growth mechanisms of the deposited metals are investigated using cyclic voltammetry (CV) and chronoamperometry (CA). Structural and electrochemical properties are characterized using X-ray diffraction, energy-dispersive X-ray spectroscopy, CV, and electrochemical impedance spectroscopy (EIS). Furthermore, the DNA sensing performance of the fabricated electrodes is evaluated through probe immobilization and hybridization detection using the [Fe(CN)_6_]^3−/4−^ redox system. This study provides an understanding of how the nucleation mechanism influences DNA biosensor sensitivity. Results demonstrate that the instantaneous nucleation behavior of Pd leads to a highly nanostructured surface and significantly enhanced DNA sensing performance compared with Au and Cu electrodes. The findings provide new insights into nucleation-controlled design strategies for high-performance electrochemical biosensors.

## 2 Experimental

### 2.1 Chemical reagents

Chemicals such as choline chloride (C_5_H_14_ClNO), urea (NH_2_CONH_2_) were obtained from Sigma-Aldrich. Potassium hexacyanoferrate(iii) (K_3_Fe(CN)_6_), potassium hexacyanoferrate(ii) (K_4_Fe(CN)_6_), tetrachloroauric acid tetrahydrate (HAuCl_4_·4H_2_O), palladium(ii) chloride (PdCl_2_), and copper chloride dihydrate (CuCl_2_2H_2_O) were purchased from Beijing Chemical Reagent (China). 3-Aminopropyl triethoxy-silance (APTES), 1-ethyl-3-dimethyl-aminopropyl carbodiimide (EDC), 1-methylimidazole (MIA), and synthetic nucleotide sequences (Table 1) were obtained from Invitrogen Co., Ltd. All chemicals were of analytical grade and used without further purification. Aqueous solutions were prepared using ultrapure deionized (DI) water with a resistivity of 18 MΩ cm.

**Table 1:** DNA strands of *M. tuberculosis* used in this work

Oligonucleotide	Sequences
DNA probe	5′-GGTCTTCGTGGCCGGCGTTCA-3′
DNA target	5′-TGAACGCCGGCCACGAAGACC-3′
Three base mismatched	5′-TGAACGCCAACCACGAAGACG-3′
Non-complementary sequence (negative control)	5′-ATGTCTCAAGCCAGCTGCTG-3′

### 2.2 Preparation of deep eutectic solvent electrolyte

Choline chloride (ChCl) and urea were mixed at a molar ratio of 1 : 2 (74.5 g of ChCl and 60.0 g of urea) and heated at 90 °C under continuous stirring for 6 h until a transparent and homogeneous deep eutectic solvent (DES) was formed. Subsequently, 0.170 g of CuCl_2_·2H_2_O, 0.394 g of HAuCl_4_·4H_2_O, and 0.177 g of PdCl_2_ were individually dissolved in the DES under stirring at 90 °C for 12 h to obtain uniform Cu^2+^, Au^3+^, and Pd^2+^ electrolytes. These electrolytes were directly employed for the electrodeposition synthesis of the corresponding metal nanostructures.

### 2.3 Electrodeposition of metals nanostructured on glassy carbon electrodes (GCEs)

A GCE was used as the working electrode for the electrochemical deposition of metal nanostructures. Prior to deposition, the electrode surface was mechanically polished with alumina slurry (0.3 and 0.05 µm), followed by ultrasonic cleaning in ethanol and DI water to remove residual particles. The GCE surface was electrochemically cleansed by CV over a potential range from −1.2 V to 2.1 V at a scan rate of 10 mV s^−1^ in 0.05 M H_2_SO_4_ solution for 5 min at room temperature. After cleaning, the electrode was rinsed thoroughly with distilled water and dried in air at room temperature. A platinum wire and an Ag/AgCl electrode were used as the counter and reference electrodes, respectively. The metal nanostructures were electrodeposited onto the GCE surface using CV. The deposition was carried out in the DES electrolyte within a potential window of −1.0 to +1.0 V at a scan rate of 50 mV s^−1^ for 10 cycles. After deposition, the metal-modified GCEs (metal/GCEs) were rinsed with DI water and dried in air prior to their use in DNA sensing experiments.

### 2.4 DNA immobilization and hybridization detection

Cu-, Au-, and Pd-modified GCEs were used as platforms for the immobilization of ssDNA probes for target DNA detection. The metal/GCEs were first modified by APTES solution to introduce amine functional groups on the electrode surface. Afterward, 0.5 × 10^−2^ M the EDC/MIA system was used to activate the phosphate groups of the DNA strands, forming reactive intermediates that subsequently react with amine groups on the APTES-functionalized electrode surface to form stable phosphoramidate linkages. The APTES-modified metal/GCEs were then modified by the activated ssDNA strands to immobilize the ssDNA probe on the electrode surface (ssDNA/metal/GCE). Finally, the ssDNA/metal/GCEs were modified using a BSA solution at room temperature.

Hybridization between the immobilized ssDNA strands and its complementary sequences was carried out by incubating the ssDNA/metal/GCEs with 20 µL of hybridization buffer containing the target DNA for 1.0 h (double-stranded DNA [dsDNA]/metal/GCEs). After hybridization, the dsDNA/metal/GCEs were thoroughly rinsed with distilled water and then dried in air conditions. Finally, the hybridization event was electrochemically detected by dipping the dsDNA/metal/GCEs in 2 mL of buffer solution containing 1.0 mM [Fe(CN)_6_]^3−/4−^ as the redox indicator.

## 3 Results and discussion

### 3.1 Electrodeposition of Pd, Au, and Cu in DES

**Fig. 1:**
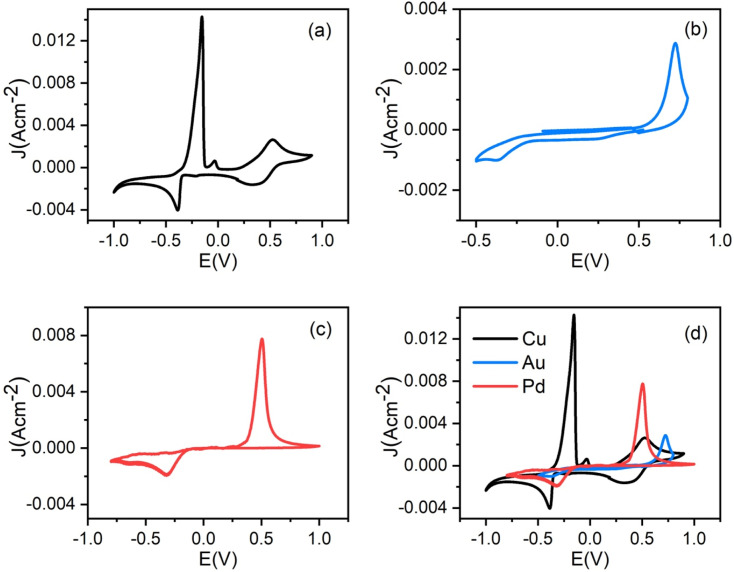
Cyclic voltammetry plots of bare GCE in choline chloride/urea (1 : 2) deep eutectic solvent with (a) Cu^2+^, (b) Au^3+^, (c) Pd^2+^ precursors and (d) comparison of CV responses for the corresponding metal modified GCEs. All measurements were conducted within a potential window of −1.0 to +1.0 V at room temperature with a scan rate of 50 mV s^−1^.

Fig. 1 indicates the CV plots of GCE in the DES with Cu^2+^, Au^3+^, and Pd^2+^ at scan rate of 50 mV s^−1^. For Cu deposition, the cathodic current peak increases sharply only at relatively negative potentials (*E*_onset_ ≈ −0.33 V), indicating that copper is not immediately deposited (Fig. 1(a)). Instead, it requires an overpotential to overcome the initial nucleation energy for nuclei formation on the electrode surface. The formed nuclei continuously grows when the current rapidly rises. By contrast, Au exhibits a more moderate cathodic response, with an onset potential of approximately −0.2 V (Fig. 1(b)). Owing to the lower nucleation barrier of Au compared with that of Cu, Au nucleation proceeds more readily. Pd demonstrates the most positive onset potential (*E*_onset_ ≈ −0.16 V) and therefore has the lowest nucleation overpotential among the three metals (Fig. 1(c)). The early rise of cathodic current suggests that Pd nuclei are formed readily under identical experimental conditions. Overall, the nucleation tendency in the DES medium follows the order Pd > Au > Cu (Fig. 1(d)), where Pd exhibits the most favorable nucleation behavior and requires the smallest overpotential for nucleus formation, whereas Cu requires the largest overpotential because of the stronger stabilization of ionic species and the higher nucleation barrier.

**Fig. 2:**
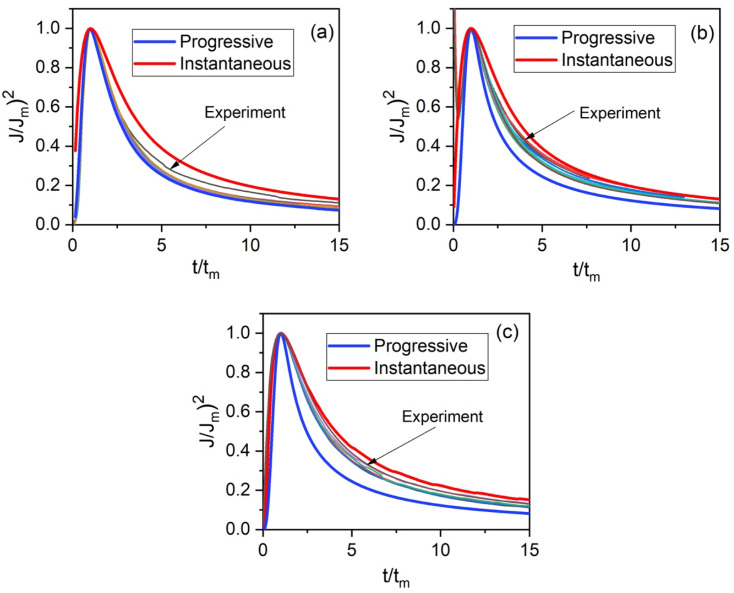
Comparison of instantaneous and progressive nucleation behaviors predicted by the Scharifker–Hills model, together with normalized experimental current density transients (*i*/*i*_m_*vs. t*/*t*_m_) for (a) Cu, (b) Au, and (c) Pd electrodeposition in choline chloride/urea (1 : 2) deep eutectic solvent.

Fig. 2 presents the dimensionless current–time transients normalized as (*J*/*J*_m_)^2^*versus t*/*t*_m_ and compared with the theoretical Scharifker–Hills (S–H) curves for 3D diffusion-controlled nucleation under two limiting cases, namely, instantaneous nucleation and progressive nucleation. As observed in Fig. 2(a), the normalized Cu transients exhibit good agreement with the progressive nucleation model, particularly in the post-maximum region (*t*/*t*_m_ > 1). This behavior indicates that Cu nuclei are generated continuously over time rather than forming simultaneously at the early stage. Such progressive nucleation suggests a relatively high nucleation barrier as mentioned in the CV curve.^36^ Therefore, Cu electrodeposition is dominated by time-dependent nucleation accompanied with subsequent 3D growth under diffusion control.

Fig. 2(b) shows the normalized Au transients lie between the two theoretical limits. At around the maximum (*t*/*t*_m_ > 1), the experimental curves show partial resemblance to the progressive model. By contrast, the decay tends to approach the instantaneous behavior at longer times. This intermediate response suggests a mixed nucleation mechanism, where an initial fraction of nuclei forms continuously over time (progressive contribution), followed by continued nucleation during rapid growth (quasi-instantaneous component). Therefore, Au deposition in DES proceeds with moderate nucleation kinetics and a balance between immediate site activation and time-dependent nucleation.

In contrast to Cu and Au, the Pd transients display the closest agreement with the instantaneous nucleation curve over a wide time domain, including the region near the maximum and the subsequent decay. This finding indicates that a large number of Pd nuclei are formed nearly simultaneously once the critical potential is reached, implying a low nucleation barrier and fast interfacial charge-transfer kinetics. The instantaneous mode is typically associated with a high density of active nucleation sites and the rapid establishment of 3D nuclei followed by diffusion-controlled growth.

The dimensionless current–time transients normalized according to the Scharifker–Hills model reveal distinct nucleation mechanisms for the three metals. Cu closely follows the progressive nucleation model, indicating that nuclei are formed continuously over time under diffusion-controlled conditions. By contrast, Pd exhibits excellent agreement with the instantaneous nucleation model, suggesting the simultaneous formation of a high density of nuclei once the critical overpotential is reached. Au displays intermediate behavior, consistent with a mixed nucleation mechanism. These findings are consistent with the CV analysis, where Pd shows the lowest nucleation overpotential and Cu displays the highest.

### 3.2 Morphology and structural characterization

**Fig. 3:**
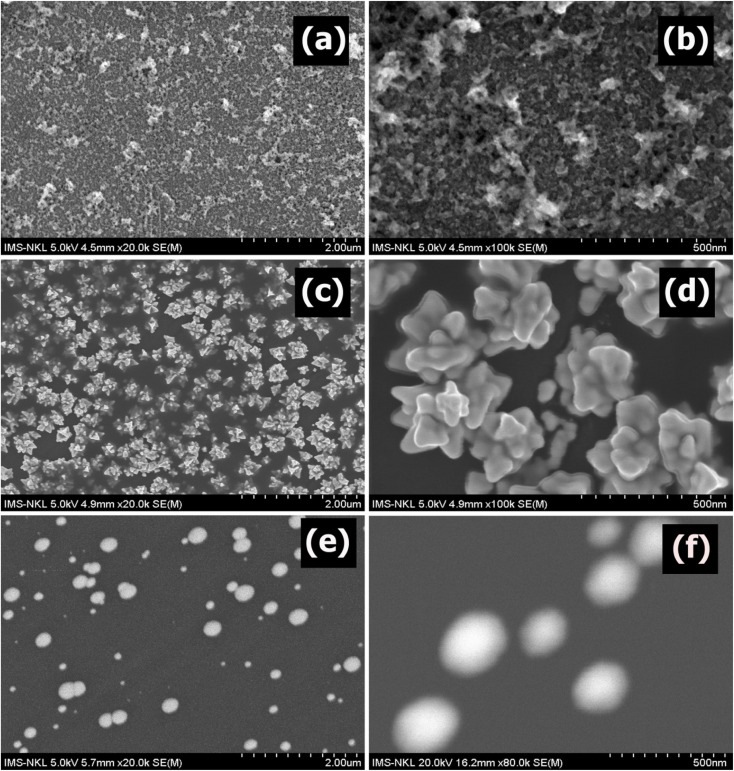
FESEM images showing the surface morphology of (a and b) Pd, (c and d) Au, and (e and f) Cu nanostructures electrodeposited on GCE from DES electrolytes using cyclic voltammetry within a potential window of −1.0 to +1.0 V at a scan rate of 50 mV s^−1^ for 10 cycles at room temperature, recorded at different magnifications.

The surface morphologies of the electrodeposited Pd, Au, and Cu films were examined by field-emission scanning electron microscopy (FESEM) as shown in Fig. 3(a–f). The FESEM images of Pd (Fig. 3(a and b)) show a highly dense distribution of Pd particles uniformly covering the electrode surface. Such morphology indicates that a large number of nuclei are generated simultaneously at the initial stage of electrodeposition. This feature is characteristic of an instantaneous nucleation mechanism, in which nucleation occurs almost simultaneously across the electrode surface once the critical overpotential is reached, resulting in a densely packed nanoparticle layer.

Au deposition (Fig. 3(c and d)) exhibit flower-like microstructures composed of aggregated nanosheets, revealing hierarchical structures formed by the assembly of small crystallites. The relatively broad size distribution and the presence of aggregated clusters suggest that nucleation and growth occur concurrently during electrodeposition. Some nuclei begin to grow while additional nuclei continue to form within a short time interval. These features are consistent with an intermediate nucleation mechanism, which lies between instantaneous and progressive nucleation.

Cu deposition (Fig. 3(e and f)) displays a markedly different morphology characterized by isolated spherical particles sparsely distributed on the electrode surface, indicating a relatively low nucleation density. This morphology suggests that the previously formed nuclei grow larger during deposition. As a result, a broad particle size distribution and low particle density are observed across the surface. Such characteristics are typical of a progressive nucleation mechanism, where nucleation occurs gradually over time rather than simultaneously.

**Fig. 4:**
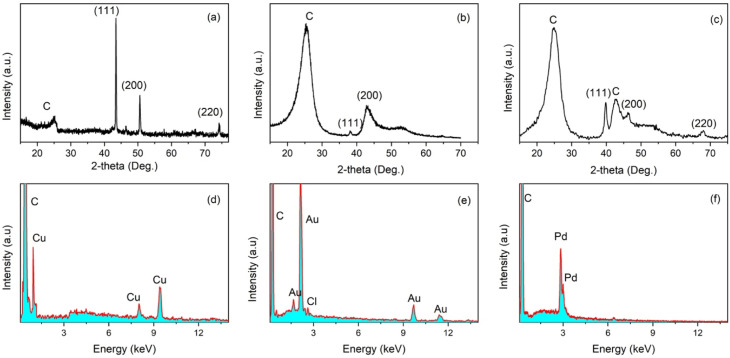
XRD patterns of (a) Cu, (b) Au, and (c) Pd and corresponding EDS spectra of (d) Cu, (e) Au, and (f) Pd nanostructures electrodeposited on GCE from choline chloride/urea (1 : 2) deep eutectic solvent using cyclic voltammetry within a potential window of −1.0 to +1.0 V at a scan rate of 50 mV s^−1^ for 10 cycles, confirming the crystalline structure and elemental composition of the deposited metals.

The crystallographic structures of the electrodeposited Cu, Au, and Pd films were examined by X-ray diffraction (XRD) as shown in Fig. 4(a–c). The XRD measurements were performed directly on the as-deposited films on the electrode surface without removing the materials, in order to preserve the structural characteristics of the nanostructures under realistic conditions.

For Cu (Fig. 4(a)), three well-defined diffraction peaks are observed at approximately 43.3°, 50.4°, and 74.1°, corresponding to the (111), (200), and (220) planes of face-centered cubic (fcc) Cu, respectively (JCPDS No. 04-0836). The highest intensity of the (111) reflection compared with other peaks (such as (200) or (220)) confirms that the majority of Cu grains are oriented with the (111) plane parallel to the surface. A broad feature at low diffraction angles is attributed to the carbon substrate of the GCE. No additional diffraction peaks are detected, confirming the phase purity of the deposited Cu film.

For Au (Fig. 4(b)), the XRD pattern shows diffraction peaks centered near 38.2° and 44.4°, corresponding to the (111) and (200) planes of fcc Au (JCPDS standard No.96-900-8464). The broad diffraction peak observed at approximately 26° corresponds to carbon of GCE. No peaks of impurities are found in the XRD pattern, emphasizing the high purity of the sample.

The XRD pattern of the electrodeposited Pd sample (Fig. 4(c)) exhibits distinct diffraction peaks centered at approximately 40.1°, 46.7°, and 68.1°, which can be indexed to the (111), (200), and (220) crystallographic planes of fcc Pd (JCPDS standard No.89-4897). A broad peak observed near ∼26° corresponds to the carbon of GCE. No additional reflections associated with PdO or other secondary phases are observed, confirming the successful deposition of crystalline metallic Pd with high purity.

The EDS spectra (Fig. 4(d–f)) further confirm the elemental composition of the electrodeposited films. For Cu (Fig. 4(d)), strong Cu signals are observed along with the carbon originating from the GCE substrate. No additional metallic impurities are detected, supporting the high purity of the deposited Cu film.

For Au (Fig. 4(e)), characteristic Au peaks are prominently detected, confirming the successful deposition of gold. In addition to Au, carbon signals from the GCE substrate are observed, together with weak chlorine (Cl) signals, which may arise from residual DES species remaining after electrodeposition. The absence of other impurity elements indicates the good compositional purity of the Au layer.

For Pd (Fig. 4(f)), the spectrum exhibits strong characteristic Pd peaks, verifying the effective reduction of Pd^2+^ ions to metallic Pd during the electrodeposition. A pronounced carbon signal is again present because of the GCE substrate. This observation is consistent with the XRD results, where a broad diffraction peak associated with carbon remains visible. No detectable signals corresponding to oxygen-rich phases or other contaminants are observed within the detection limit of EDS.

### 3.3 Electrochemical characterization of the metal/GCE by CV and EIS

**Fig. 5:**
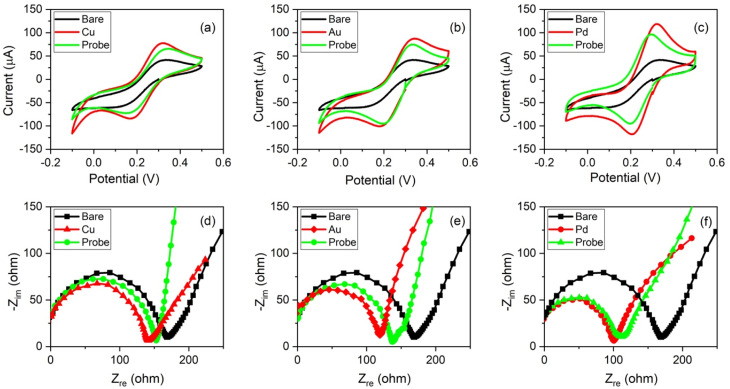
CV responses of bare GCE, metal-modified GCE, and probe DNA-modified electrodes are shown for (a) Cu, (b) Au, and (c) Pd. Corresponding Nyquist plots are presented in (d) Cu, (e) Au, and (f) Pd. Measurements were carried out in 0.1 M PBS (pH 7.4) containing 1.0 mM [Fe(CN)_6_]^3−/4−^ at room temperature. CV was recorded from −0.1 to +0.5 V at a scan rate of 100 mV s^−1^, and EIS measurements were performed over the frequency range of 0.1 Hz–100 kHz with an AC perturbation amplitude of 5 mV.

The electrochemical properties of the Cu-, Au-, and Pd-modified electrodes were evaluated using CV and EIS in a buffer solution containing the redox probe [Fe(CN)_6_]^3−/4−^ as presented in Fig. 5.

Fig. 5(a–c) show the CV responses of the bare electrode, metal-modified electrodes (Cu, Au, and Pd), and ssDNA-modified electrodes. Among the three systems, the bare electrode exhibits relatively small redox currents (41.68 µA), indicating limited electron transfer between the electrode surface and the [Fe(CN)_6_]^3−/4−^ redox couple. After the electrodeposition of Cu, Au, and Pd, the peak currents increase significantly compared with that of the bare electrode (78.88 µA for Cu, 87.1 µA for Au, and 119.52 µA for Pd). This phenomenon can be attributed to the significantly increased electroactive surface area of the electrode, providing additional active sites for the redox reaction of the [Fe(CN)_6_]^3−/4−^ probe.^37^ According to the Randles–Sevcik equation,^38^ the peak current is directly proportional to the effective electrode area, leading to high current responses for the metal-modified electrodes. The electroactive surface areas of the electrodes were estimated using the Randles–Sevcik equation based on the anodic peak currents of the [Fe(CN)_6_]^3−/4−^ redox probe at a scan rate of 100 mV s^−1^. The calculated effective surface areas are 0.371, 0.69, 0.783, and 1.049 cm^2^ for the bare, Cu-, Au-, and Pd-modified electrodes, respectively. The Pd-modified electrode exhibits the largest effective surface area, which can be attributed to its instantaneous nucleation mechanism that produces a high density of nuclei. The Au-modified electrode shows an intermediate surface area ascribed to its mixed nucleation behavior, while Cu-modified electrode presents the smallest surface area caused by progressive nucleation, which leads to large crystallite growth. These results are consistent with the nucleation analysis and structural characterization discussed above.

The roughness factor (RF) of the modified electrodes was estimated by comparing their electroactive surface area with that of the bare electrode. The calculated RF values are 1.86, 2.11, and 2.83 for the Cu-, Au-, and Pd-modified electrodes, respectively. The significantly higher RF of the Pd-modified electrode indicates that its real surface area is larger than its geometric surface area. Such an increase in surface roughness is typically associated with the formation of nanostructured surfaces that provide a high density of electrochemically active sites.^39,40^ This result is consistent with the instantaneous nucleation mechanism of Pd, that is, numerous nuclei are generated simultaneously during electrodeposition. By comparison, the Au-modified electrode exhibits a moderate RF, which can be attributed to its mixed nucleation behavior, resulting in an intermediate density of surface nanostructures. The Cu-modified electrode shows the lowest RF because of its progressive nucleation mechanism, which favors the growth of large crystallites and consequently reduces the density of active surface sites. These findings further confirm the strong correlation among nucleation mechanism, surface morphology, and electrochemical activity.

After the immobilization of the DNA probe, the peak current decreases significantly compared with those of the metal-modified electrodes (65.86 µA for Cu, 75.14 µA for Au, and 95.31 µA for Pd). This reduction can be attributed to the formation of a negatively charged DNA layer on the electrode surface. The phosphate backbone of DNA carries negative charges, which electrostatically repel the negatively charged [Fe(CN)_6_]^3−/4−^ redox probe, thereby hindering its diffusion toward the electrode surface. In addition, the immobilized DNA layer acts as a partially insulating barrier that increases the charge-transfer resistance at the electrode–electrolyte interface.^41^ As a result, the electron-transfer becomes less efficient, leading to a decrease in the redox peak currents.^42^ The reduction in peak current therefore confirms the successful immobilization of probe molecules on the electrode surface.

The EIS results shown in Fig. 5(d–f) further support these observations. The Nyquist plots consist of a semicircular region at high frequency, followed by a linear diffusion tail at low frequency corresponding to charge transfer and diffusion processes. The charge-transfer resistance (*R*_ct_) can be estimated from the diameter of the semicircle. For the bare electrode, the *R*_ct_ is approximately 171 Ω. After metal deposition, the semicircle diameter decreases markedly, indicating improved interfacial electron-transfer kinetics. The estimated *R*_ct_ values are 142 Ω for Cu, 119 Ω for Au, and 99 Ω for Pd, demonstrating that Pd provides the lowest charge-transfer resistance among the three metals. After probe immobilization, the *R*_ct_ values increase again to approximately 154 Ω for Cu/ssDNA, 140 Ω for Au/ssDNA, and 110 Ω for Pd/ssDNA because of the blocking effect of the probe layer, electrostatically repelling the interaction between the negatively charged [Fe(CN)_6_]^3−/4−^ redox probe and DNA. Nevertheless, the Pd-based electrode still exhibits the smallest *R*_ct_, confirming its superior electron-transfer capability. This result is fully consistent with the CV data and the large electroactive surface area and roughness factor of Pd, indicating that Pd forms a highly conductive nanostructured surface with abundant electroactive sites.

### 3.4 Electrochemical detection performance of the DNA sensors

**Fig. 6:**
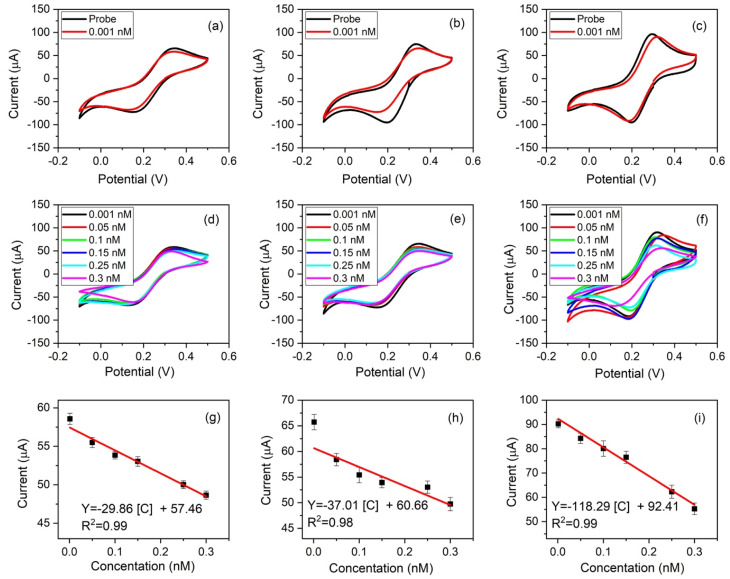
Electrochemical DNA sensing performance of Cu-, Au-, and Pd-based electrodes. Cyclic voltammetric responses recorded before and after hybridization with complementary target DNA (0.001 nM) for (a) Cu-, (b) Au-, and (c) Pd-based sensors in the presence of 1.0 mM [Fe(CN)_6_]^3−/4−^. Concentration-dependent CV responses toward target DNA (0.001–0.3 nM) are shown in (d) Cu-, (e) Au-, and (f) Pd-based sensors. The corresponding calibration curves are presented in (g) Cu-, (h) Au-, and (i) Pd-based sensors. Measurements were carried out in 0.1 M PBS (pH 7.4) within a potential window of −0.1 to +0.5 V at a scan rate of 100 mV s^−1^.

The electrochemical performance of the DNA sensors based on ssDNA/Cu-, ssDNA/Au-, and ssDNA/Pd-modified electrodes in the presence of the [Fe(CN)_6_]^3−/4−^ redox probe was evaluated by CV. Fig. 6(a–c) show the CV responses of the ssDNA/metal-modified electrodes before and after hybridization with target DNA at a concentration of 0.001 nM for (a) Cu, (b) Au, and (c) Pd. After hybridization, a noticeable decrease in the peak current is observed for all three electrodes. This decrease is attributed to the formation of dsDNA on the electrode surface, which creates a negatively charged and partially insulating layer. The phosphate backbone of DNA electrostatically repels the negatively charged [Fe(CN)_6_]^3−/4−^ redox probe redox species, thereby hindering electron transfer at the electrode–electrolyte interface.

Among the three electrodes, the Pd-based sensor exhibits the most significant current decrease after hybridization (10.02%), showing the most pronounced blocking effect caused by DNA duplex formation. This observation is consistent with the significantly larger electroactive surface area (1.049 cm^2^) and roughness factor (2.83) of the Pd electrode compared with Au and Cu. The highly nanostructured Pd surface provides a large number of electroactive sites and improved electron-transfer properties, which provide favorable conditions for probe DNA immobilization and facilitate efficient target DNA hybridization.

The Au-based sensor exhibits a moderate current decrease after hybridization compared with the Pd electrode (8.15%). This behavior can be attributed to the intermediate electroactive surface area (0.783 cm^2^) and roughness factor (2.11) of the Au electrode, which provide a moderate number of active sites for probe DNA immobilization. In addition, the mixed nucleation mechanism of Au leads to a density of nanoscale particles compared with the instantaneous nucleation behavior observed for Pd, resulting in a lower density of immobilized probe DNA strands.

The Cu electrode shows the smallest current variation after hybridization (6.23%). This result can be explained by its lowest electroactive surface area and roughness factor, which limit the number of probe DNA strands that can be immobilized on the electrode surface. Furthermore, the progressive nucleation mechanism of Cu typically results in large crystallites and a low density of nanostructures, thereby reducing the available active sites for DNA hybridization. Consequently, the hybridization efficiency and sensitivity of the Cu-based sensor are lower than those of the Au and Pd electrodes.

The nucleation mechanism plays a crucial role in determining the surface morphology and ultimately the biosensing performance of the electrode. In this study, Pd follows an instantaneous nucleation mechanism, Cu exhibits progressive nucleation, and Au shows intermediate behavior. In instantaneous nucleation, a large number of nuclei are generated simultaneously once the critical overpotential is reached. This process produces a high density of uniformly distributed nanoscale particles with relatively small particle size, resulting in enhanced surface roughness and abundant accessible immobilization sites for probe DNA.

A large electroactive surface area provides abundant active sites for probe DNA immobilization and improves interfacial electron-transfer behavior. These characteristics create favorable conditions for efficient target DNA hybridization and enhance the blocking effect on electron transfer between the electrode surface and the [Fe(CN)_6_]^3−^/^4−^ redox probe, resulting in a decrease in the electrochemical current. The electrochemical response is influenced by the combined effects of surface morphology, probe immobilization behavior, interfacial charge-transfer characteristics, and DNA-induced blocking effects. By contrast, progressive nucleation, as observed for Cu, leads to the gradual formation and growth of larger crystallites with lower nuclei density and less uniform particle distribution. These structural characteristics reduce the number of accessible immobilization sites and may result in less favorable probe DNA distribution and reduced target accessibility during hybridization. Consequently, the hybridization efficiency and blocking effect are lower than those observed for the Pd electrode. Meanwhile, the Au electrode shows intermediate behavior because of its mixed nucleation mechanism and intermediate surface characteristics.

Therefore, the electrochemical DNA sensing performance is governed not only by electroactive surface area, but also by nucleation-induced variations in nuclei density, particle-size distribution, nanostructure uniformity, probe immobilization behavior, target accessibility, interfacial electron-transfer characteristics, and DNA-induced blocking effects.

Fig. 6(d–f) displays the CV responses of the sensors at different target DNA concentrations ranging from 1.0 × 10^−12^ M to 3.0 × 10^−10^ M. As the concentration of target DNA increases, the peak current gradually decreases for all electrodes because of the increasing surface coverage of hybridized DNA, which further blocks electron transfer between the electrode surface and the redox probe. The electrochemical responses are influenced by the combined effects of surface morphology, electroactive surface area, probe DNA immobilization behavior, and interfacial blocking effects induced by DNA hybridization. Among the investigated electrodes, the Pd-modified electrode exhibits favorable electrochemical characteristics, including large electroactive surface area and enhanced interfacial electron-transfer behavior, which provide suitable conditions for probe DNA immobilization and DNA hybridization.

The calibration curves derived from the peak current responses are presented in Fig. 6(g–i). A linear relationship between the peak current and target DNA concentration is observed within the tested concentration range for all sensors. The regression equations are obtained as *Y* = −29.86[*C*] (nM) + 57.46 for Cu (*R*^2^ = 0.99), *Y* = −37.01[*C*] (nM) + 60.66 for Au (*R*^2^ = 0.98), and *Y* = − 118.29[*C*] (nM) + 92.41 for Pd (*R*^2^ = 0.99). The obtained sensitivity values are 80.48, 163.35, and 318.54 µA nM^−1^ cm^−2^ for Cu, Au, and Pd, respectively.

According to the signal-to-noise ratio criterion (S/N = 3), the limits of detection (LODs) of the sensors are 1.97 × 10^−12^ M, 1.17 × 10^−12^ M, and 0.73 × 10^−12^ M for Cu, Au, and Pd, respectively. Overall, the results indicate that the Pd-based sensor provides the highest sensitivity and lowest detection limit for DNA detection, highlighting the advantage of Pd nanostructures in electrochemical biosensing applications.

For further evaluation of the performance of the developed sensors, a comparison with previously reported electrochemical DNA sensors for *Mycobacterium tuberculosis* detection is presented in Table 2.

**Table 2:** Comparison of the performance of electrochemical DNA sensors for *M. tuberculosis* detection

Electrode material	Detection method	Linear range (M)	LOD (M)	Sensitivity (µA nM^−1^ cm^−2^)	Reference
Au/SPE^a^	DPV^b^	2.0 × 10^−9^ to 10.0 × 10^−9^	1.9 × 10^−9^	Not reported	12
PANI/GP^c^ composite	DPV	10^−9^ to 10^−6^	7.853 × 10^−7^	Not reported	43
Au electrode	SWV^d^	Not reported	14.5 × 10^−9^	Not reported	14
PGA^e^/PGE^f^	SWV	1.5 × 10^−9^ to 12.5 × 10^−9^	1.3 × 10^−9^	Not reported	44
MPA^g^-Fe_3_O_4_/NCC^h^/CTAB^i^/SPE	DPV	1.0 × 10^−12^ to 1.0 × 10^−6^	7.96 × 10^−13^	Not reported	45
Cu/GCE^j^	CV^k^	1.0 × 10^−12^ to 3.0 × 10^−10^	1.97 × 10^−12^	80.48	This work
Au/GCE	CV	1.0 × 10^−12^ to 3.0 × 10^−10^	1.17 × 10^−12^	163.35	This work
Pd/GCE	CV	1.0 × 10^−12^ to 3.0 × 10^−10^	0.73 × 10^−12^	318.54	This work

a SPE: screen-printed electrodes.

b DPV: differential pulse voltammetry.

c PANI/GP: polyaniline/graphene.

d SWV: squarewave voltammetry.

e PGA: poly(l-glutamic) acid.

f PGE: pencil graphite electrode.

g MPA: mercaptopropionic acid.

h NCC: nanocellulose crystalline.

i CTAB: cetyl trimethyl ammonium bromide.

j GCE: glassy carbon electrode.

k CV: cyclic voltammetry.

As shown in Table 2, the developed DNA sensors in this work exhibits a competitive detection limit and a wide linear detection range compared with previously reported sensors. In particular, the Pd-modified electrode demonstrates the highest sensitivity among the three tested materials, which can be attributed to its instantaneous nucleation mechanism that leads to a high-density nanostructured surface. The findings demonstrate that the Pd-based sensor achieves excellent analytical performance while maintaining a straightforward fabrication process and reasonable cost, highlighting its potential for reliable and practical electrochemical DNA sensing applications.

### 3.5 Reproducibility, stability, and selectivity of DNA biosensor

The practical applicability of the fabricated DNA biosensors was further evaluated in terms of reproducibility, stability, and selectivity.

**Fig. 7:**
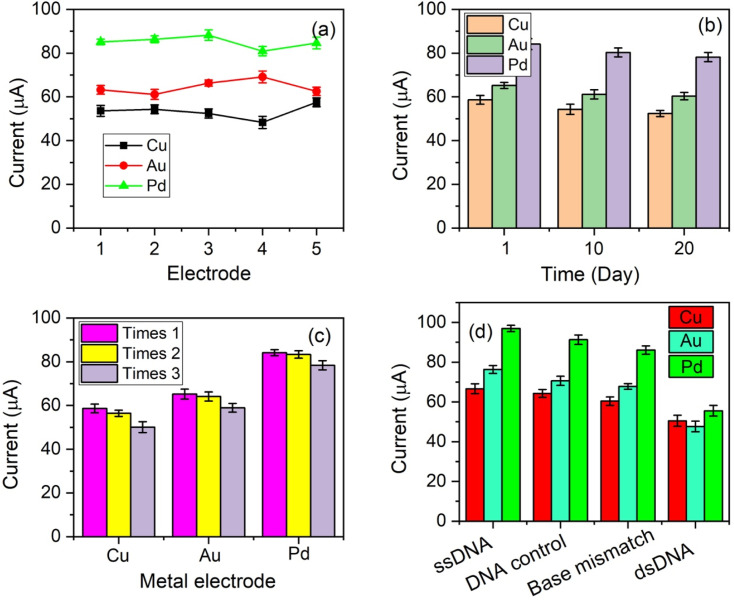
(a) Reproducibility of the DNA sensors evaluated using five independently prepared electrodes. (b) Storage stability of the sensors, assessed by measuring the current response after storage in PBS at 4 °C for 1, 10, and 20 days. (c) Operational stability of the sensors examined through repeated hybridization–dehybridization cycles. (d) Selectivity of the sensors toward different DNA sequences including ssDNA probe, complementary target DNA (dsDNA), three-base mismatched sequences, and noncomplementary sequences (DNA control) under identical conditions. Error bars represent the standard deviation of three independent measurements (*n* = 3).

The reproducibility of the sensors was investigated by measuring the electrochemical response of five independently prepared electrodes under identical experimental conditions. Reproducibility was tested by measuring the change in peak currents as shown in Fig. 7(a). The relative standard deviation (RSD) values of the peak current responses are 3.34% for Cu, 3.22% for Au, and 2.68% for Pd, indicating good fabrication reproducibility. Among them, the Pd-based electrode exhibited the lowest RSD, which can be attributed to its uniform nanostructured surface formed *via* instantaneous nucleation, leading to consistent probe immobilization and electron-transfer characteristics.

The stability of the DNA biosensors was evaluated by storing the ssDNA-modified metal/GCE at 4 °C and measuring their electrochemical responses over time. Fig. 7(b) shows that after 10 days, the sensors retain approximately 92.66% (Cu), 93.71% (Au), and 95.48% (Pd) of their initial current response. After 20 days, the retained signals decreased to 88.9% (Cu), 90.95% (Au), and 92.98% (Pd) of their initial current response. The Au- and Pd-based sensors show higher signal retention compared with the Cu-based sensor after 20 days, indicating their good stability. This enhanced stability can be attributed to the strong adhesion of Au and Pd nanostructures to the electrode surface and their high resistance to surface oxidation. By contrast, the Cu-based electrode shows relatively low stability, which is likely due to partial oxidation and surface degradation over prolonged storage.

The stability of the sensors was further evaluated through repeated hybridization–dehybridization cycles between ssDNA and dsDNA. As shown in Fig. 7(c), the hybridization response after two cycles exhibits negligible changes for all sensors, indicating good operational stability. After the third hybridization cycle, the signal variation relative to the initial response is approximately 14.5% (Cu), 9.66% (Au), and 6.67% (Pd). These results demonstrate that the DNA sensors possess acceptable stability, with the Pd-based sensor showing the highest signal retention during repeated use, reflecting its interfacial stability.

The selectivity of the developed DNA sensors was evaluated using different DNA sequences, including complementary target DNA (dsDNA), three-base mismatched sequences, and noncomplementary strands (DNA control) under identical conditions. As illustrated in Fig. 7(d), the complementary target DNA induces a pronounced decrease in peak current, the mismatched sequences produce only minor changes in the signal, and the noncomplementary sequence causes negligible variation in the current response. These results demonstrate that the sensor can effectively discriminate among perfectly matched, mismatched, and noncomplementary sequences. Among the three electrodes, the Pd-based sensor exhibits the highest selectivity, showing the largest signal difference between complementary and mismatched sequences. This superior performance can be attributed to its high electroactive surface area and the dense distribution of active sites, which promote efficient probe immobilization and hybridization specificity.

### 3.6 Real sample analysis

**Fig. 8:**
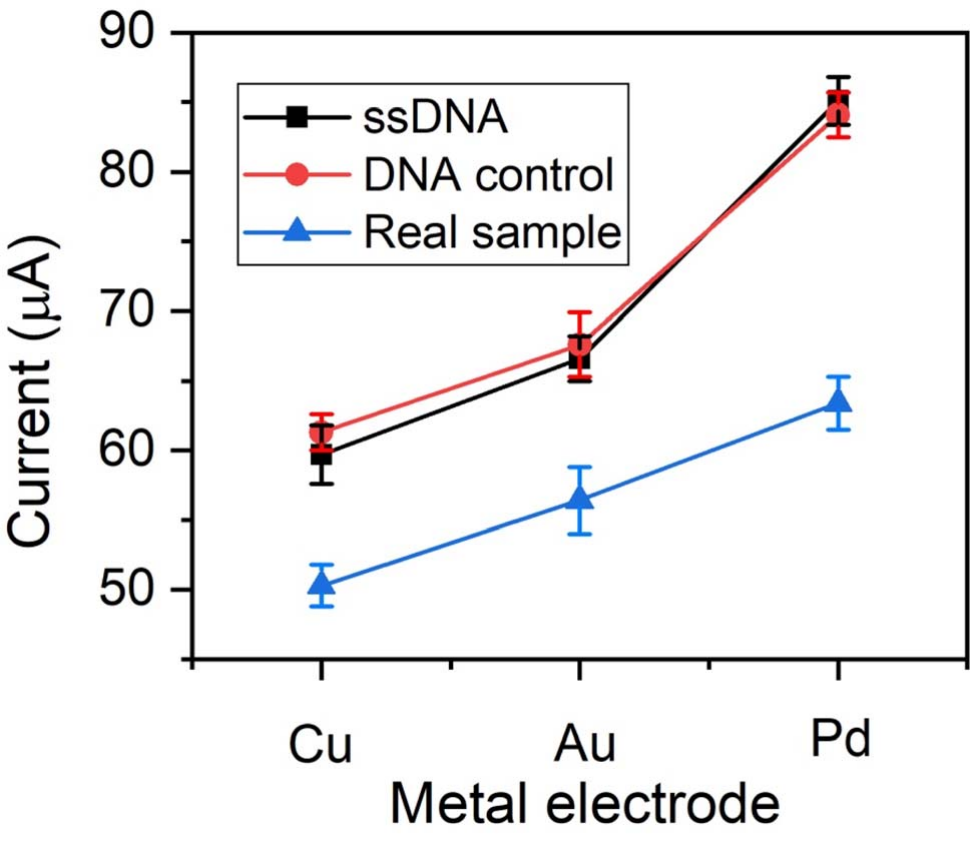
Peak current responses of Cu-, Au-, and Pd-based DNA sensors after hybridization with DNA extracted from sputum samples, compared with ssDNA-modified electrodes and noncomplementary control sequences. Error bars represent standard deviations from three independent measurements (*n* = 3).

The Cu-, Au-, and Pd-based sensors were applied to detect target DNA extracted from sputum samples of patient with tuberculosis to evaluate the practical applicability of the developed DNA biosensors. As shown in Fig. 8, the peak current of metal-modified electrodes for the negative control detection changes negligible relative to the ssDNA-modified electrode, indicating minimal nonspecific interaction. By contrast, a significant decrease in current is observed for the those electrodes after hybridization with DNA extracted from *M. tuberculosis*-positive samples, with values of 50.53 µA for Cu, 56.4 µA for Au, and 63.4 µA for Pd. This decrease can be attributed to the formation of dsDNA, which introduces a negatively charged and partially insulating layer that hinders electron transfer due to electrostatic repulsion with the negatively charged [Fe(CN)_6_]^3−/4−^ redox probe. Among the tested electrodes, the Pd-based sensor exhibits the most pronounced current decrease, indicating its superior hybridization efficiency and higher sensitivity compared with Au and Cu electrodes. This enhanced performance highlights the strong potential of the Pd-based sensor for the reliable detection of DNA sequences derived from PCR-amplified sputum samples.

Recovery experiments were conducted by spiking known concentrations of target DNA into real sputum samples to evaluate the analytical accuracy of the developed sensors in complex biological matrices (Table 3). Following sample pretreatment, DNA extraction, PCR amplification, and thermal denaturation, the spiked samples were subjected to the same hybridization and electrochemical detection procedures as described above. The concentration of target DNA was determined from the corresponding calibration curve, and the recovery was calculated as follows:
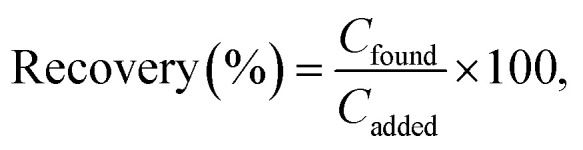
where *C*_found_ and *C*_added_ represent the measured concentration after spiking concentration in the real sample and the added concentration of target DNA, respectively.

**Table 3:** Recovery and RSD value results for metal-based DNA sensors in sputum sample (*n* = 3)

Metal	Sample	Added (nM)	Found (nM)	Recovery (%)	RSD (%)
Pd	Sputum 1	0.005	0.0050	100.88	3.84
		0.050	0.0484	96.82	3.98
		0.100	0.1058	105.76	3.63
Au	Sputum 1	0.005	0.0050	100.51	4.64
		0.050	0.0462	92.41	5.05
		0.100	0.0953	95.29	4.8
Cu	Sputum 1	0.005	0.0048	96.00	7.23
		0.050	0.0476	95.11	6.83
		0.100	0.1036	103.59	5.95

For the Pd-based sensor, the recovery values range from 96.82% to 105.76%, with the RSD below 3.98%, indicating excellent analytical performance. The Au-based sensor exhibits recovery values in the range of 92.41% to 100.51%, with RSD below 5.05%, demonstrating acceptable analytical performance. The Cu-based sensor showed recovery values between 95.11% and 103.59%, with slightly higher RSD (up to 7.23%), suggesting its relatively lower precision compared with Pd and Au. These results demonstrate that the developed sensors, particularly the Pd-based electrode, possess good accuracy, reliability, and applicability for DNA detection in complex real samples.

## 4 Conclusions

A systematic comparative study of Cu-, Au-, and Pd-based nanostructured electrodes synthesized in a DES was conducted to elucidate the relationship between nucleation behavior and electrochemical DNA sensing performance. Results demonstrate that the sensing performance strongly depends on the nucleation mechanism and follows the order: Pd (instantaneous) > Au (mixed) > Cu (progressive). Among the investigated electrodes, the Pd-based sensor exhibits the highest analytical performance with a sensitivity of 318.54 µA nM^−1^ cm^−2^, a detection limit of 0.73 × 10^−12^ M, and a wide linear detection range from 1.0 × 10^−12^ to 3.0 × 10^−10^ M. The developed sensors demonstrate excellent reproducibility (RSD < 3.2%), good stability, and high selectivity toward complementary DNA sequences. The practical applicability of the sensors was also validated through the detection of PCR-amplified DNA extracted from sputum samples of patients with tuberculosis, showing satisfactory recovery (92.41–105.76%) and low RSD values (<7.23%), confirming their reliability in complex biological matrices. This study established a direct correlation between nucleation behavior and DNA sensing performance, providing new insights into the design of high-performance electrochemical biosensors. The results highlighted the potential of fabricating advanced biosensing platforms for clinical and environmental applications.

## Conflicts of interest

There are no conflicts to declare.

## Supplementary Material

RA-OLF-D6RA02774A-s001

## Data Availability

The authors confirm that the data supporting the findings of this study are available within the article and its supplementary information (SI). Additional data are available from the corresponding author upon reasonable request. Supplementary information is available. See DOI: https://doi.org/10.1039/d6ra02774a.
