# Supplementary material for: Comparative study of Pd-, Au-, and Cu-based nanostructured electrodes synthesized in deep eutectic solvents: linking nucleation mechanisms to electrochemical DNA sensing performance
Source: RSC Adv. 2026 Jul 2;16(34):33156–69. doi: 10.1039/d6ra02774a (PMC13326669; doi:10.1039/d6ra02774a)
Supplement: RA-016-D6RA02774A-s001 [file RA-016-D6RA02774A-s001.pdf]

Supplementary Information (SI) for RSC Advances.
This journal is © The Royal Society of Chemistry 2026

Supporting Information

Comparative study of Pd-, Au-, and Cu-based nanostructured electrodes synthesized in deep eutectic solvents: Linking nucleation mechanisms to electrochemical DNA sensing performance

Phuong Dinh Tam[1*], Dao Vu Phuong Thao[1], Nhi Phuong Hien[2], Dinh Van Tuan[3], Nguyen Thi Nguyet[4]

[1]Faculty of Materials Science and Engineering, Phenikaa School of Engineering, Phenikaa University, Hanoi, Vietnam

[2] Department of Chemistry, Hartwick College, Oneonta, NY 13820

[3]Electric Power University, Hanoi, Viet Nam

[4]Faculty of Chemical and Environment, Hung Yen University of Technology and Education, Hung Yen, Viet Nam

*Corresponding author

Phuong Dinh Tam, Professor

Faculty of Material Science and Engineering, Phenikaa School of Engineering, Phenikaa University

E-mail: tam.phuongdinh@phenikaa-uni.edu.vn or phuongdinhtam@gmail.com

Postal address: Hanoi, 10000, Vietnam

1. Electrochemical surface area and Roughness factor calculations

The roughness factor (RF) was calculated using the ratio between the electroactive surface area (ECSA) and the geometric area of the bare electrode according to [1]:

$$RF = \frac{A_{ECSA}}{A_{geo}} \quad (1),$$

where A_{ECSA} is the electroactive surface area obtained from the Randles–Sevcik equation and A_{geo} is the geometric area of the bare glassy carbon electrode (GCE).

The ECSA values were estimated using the Randles–Sevcik equation [2]:

$$I_P = 2.69 \times\ 10^5 n^{2/3} A D^{1/2} S v^{1/2} \quad (2)$$

where Ip is the peak current, n is the number of electrons transferred, S is the electroactive surface area, D is the diffusion coefficient, C is the concentration of the redox probe, and v is the scan rate.

To further clarify this point, the corresponding explanation and supporting references have been added to the revised manuscript.

Reference:

1. A. Sukeri, A facile electrochemical approach to fabricate a nanoporous gold film electrode and its electrocatalytic activity towards dissolved oxygen reduction, Phys. Chem. Chem. Phys., vol. 17, pp. 28510-28514, Sep. 2015, https://doi.org/10.1039/C5CP05220C
2. T.Van, High-performance nonenzymatic electrochemical glucose biosensor based on AgNP-decorated MoS2 microflowers, Curr. Appl. Phys., vol. 43, pp.116-123, Nov.2022, https://doi.org/10.1016/j.cap.2022.09.001

2. Role of DES electrolyte on nucleation behavior

The choline chloride/urea-based deep eutectic solvent (DES) was selected because of its wide electrochemical potential window, high ionic conductivity, low vapor pressure, and excellent thermal stability, which are favorable for controlled electrodeposition of metallic nanostructures. In addition, the strong hydrogen-bonding network formed between choline chloride and urea influences ion diffusion and crystal growth during electrodeposition. Compared with conventional aqueous electrolytes, the relatively high viscosity and reduced ion mobility of the DES slow down the diffusion process and promote more controllable nucleation and growth behavior. As a result, different metals exhibit distinct nucleation mechanisms, nuclei density, and particle-size distribution in the DES electrolyte, which subsequently influence surface morphology, interfacial electron-transfer characteristics, probe DNA immobilization behavior, and electrochemical sensing performance. Specifically, Pd tends to exhibit instantaneous nucleation in the DES electrolyte, producing a high density of uniformly distributed nanoscale nuclei, whereas Cu shows progressive nucleation with larger and less uniformly distributed particles. Au exhibits intermediate behavior because of its mixed nucleation mechanism.

3. Design of primers

Table S1. Primer sequences used for PCR amplification

Oligonucleotide	Sequences
Forward primer	5'-AGCTGACCTGATCGGTCTTC-3'
Reverse primer	5'-CAGTTCGATGACGGTTCACT-3'

4. Sample pretreatment, DNA extract and PCR amplification

Sputum samples were collected from tuberculosis (TB) patients following standard clinical procedures. Prior to analysis, the samples were pretreated to remove mucus and cellular debris. Briefly, 500 μL of sputum sample was transferred into a tube and mixed with a 4% NaOH solution. The mixture was vortexed thoroughly and incubated at 37 °C for 15 min to achieve homogenization. Subsequently, the samples were centrifuged at 3000 rpm for 15 min. The supernatant was carefully discarded, and the resulting pellet was collected and washed with phosphate-buffered saline (PBS) to remove residual NaOH.

For DNA extraction, the pellet was resuspended in lysis buffer, followed by the addition of Proteinase K and incubation at 56 °C for 30 min to ensure complete disruption of cell membranes. The mixture was then then purified using a silica-based spin column according to standard protocols, including washing and elution steps to obtain purified genomic DNA.

Polymerase chain reaction (PCR) was performed to amplify the target DNA sequence. The PCR mixture (25 μL total volume) consisted of 12.5 μL of 2× PCR Master Mix, 1 μL of forward primer (10 μM), 1 μL of reverse primer (10 μM), 2 μL of DNA template, and 8.5 μL of nuclease-free water. The amplification was carried out under the following thermal cycling conditions: initial denaturation at 95 °C for 5 min, followed by 35 cycles of denaturation at 95 °C for 30 s, annealing at 58–60 °C for 30 s, and extension at 72 °C for 30 s, with a final extension at 72 °C for 5 min. The real sputum-derived DNA samples used for the hybridization experiments were obtained after PCR amplification and thermal denaturation. The resulting PCR products were directly used for hybridization without additional dilution.

5. Electrochemical detection in real samples

The PCR-amplified DNA samples were diluted in Tris–HCl buffer and thermally denatured at 98 °C for 5 min, followed by rapid cooling in an ice bath for 2 min to obtain single-stranded DNA prior to hybridization. For the hybridization step, 10 μL of the extracted DNA samples was carefully dropped onto the ssDNA-modified electrode surface and incubated at room temperature for 60 min at room temperature. After incubation, the electrodes were thoroughly rinsed with deionized water to remove non-specifically adsorbed DNA molecules. Electrochemical measurements were subsequently carried out in 2 mL of phosphate-buffered saline (PBS) containing 1.0 mM $[Fe(CN)_6]^{3-/4-}$ as the redox probe using cyclic voltammetry.

6. Agorose gel electrophoresis of the PCR product amplified in the thermal cycler

Although PCR was employed in this study as a target DNA amplification step to obtain sufficient DNA concentration from sputum samples, the final identification of the amplified DNA was performed using the developed electrochemical DNA sensors. In clinical practice, PCR and real-time PCR are considered standard methods for tuberculosis DNA detection because of their excellent sensitivity and specificity. However, these techniques generally require expensive thermal cyclers, fluorescence detection systems, skilled operators, and well-equipped laboratory facilities. In contrast, electrochemical DNA sensors offer several practical advantages, including lower instrumentation cost, simpler operation, rapid signal acquisition, portability, and greater potential for miniaturization and point-of-care deployment. Therefore, the combination of PCR amplification and electrochemical transduction represents a promising alternative strategy for sensitive and cost-effective nucleic acid detection, particularly in resource-limited settings.
